# Interplay of Stereoelectronic and Vibrational Modulation Effects in Tuning the UPS Spectra of Unsaturated Hydrocarbon Cage Compounds

**DOI:** 10.1021/acs.jctc.0c00645

**Published:** 2020-07-15

**Authors:** Lorenzo Paoloni, Marco Fusè, Alberto Baiardi, Vincenzo Barone

**Affiliations:** † Scuola Normale Superiore, Piazza dei Cavalieri 7, 56125 Pisa, Italy; ‡ Lab. für Physikalische Chemie, Vladimir-Prelog-Weg 1-5/10, 8093 Zürich, Switzerland

## Abstract

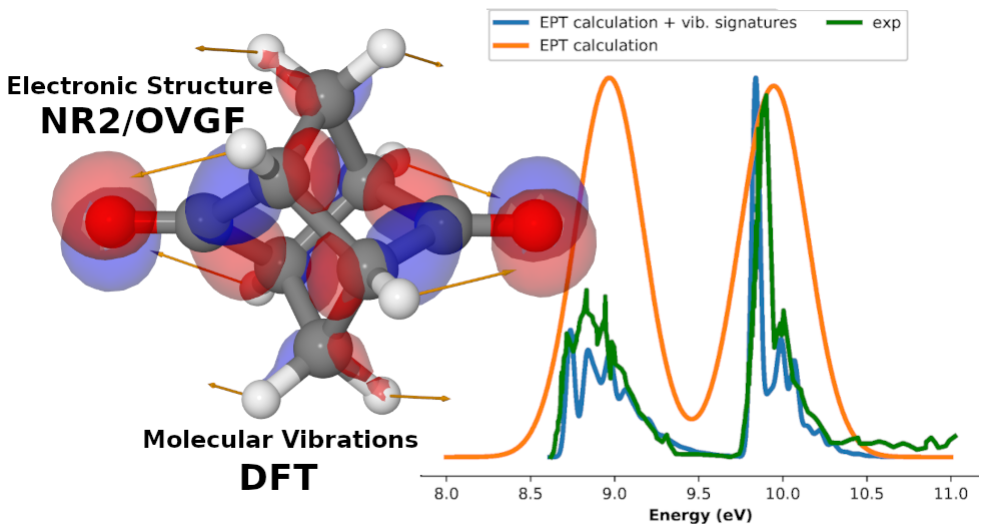

The UPS spectra of six hydrocarbon cage compounds have been investigated by a Green-function approach in conjunction with a full harmonic treatment of vibrational modulation effects. The remarkable agreement with experimental results points out the reliability of the proposed computational approach and the strong interplay of stereoelectronic and vibrational effects in tuning the overall spectra.

## 1 Introduction

The interactions between π-bonds tuned by a rigid σ-scaffold are of considerable interest especially in connection with the fine-tuning of long-range electron- and energy-transfer between chromophores.^1^ While it is well-known that the rate of through-space electron transfer decreases exponentially with the donor–acceptor distance,^2−5^ when this distance exceeds about 3 Å, the strength of the through-bond coupling shows a remarkable sensitivity to the detailed structure of the rigid σ-scaffold.^6,7^ This has stimulated a number of experimental studies aimed at rationalizing the behavior of known compounds and to design new purposely tailored molecular systems. In this connection, one of the methods of choice for investigating the strength of this kind of interactions is ultraviolet (He I) photoelectron spectroscopy (UPS).^8,9^

UPS spectroscopy provides useful experimental data related to the structure of neutral molecular systems and their ionized counterparts: pieces of information about the electronic structures of the neutral and ionized forms of the molecule (as well as about nuclear dynamics and electronic structures) are intertwined in the experimental data, so that general, reliable, and robust computational tools provide an invaluable support for rationalization and analysis of experimental results. Thanks to Koopmans’ theorem,^10^ which relates the molecular ionization energies to the energy of the orbital from which the photoionized electron is removed, UPS is commonly employed to characterize outer-valence molecular orbitals. Nonetheless, when vibrational progressions are resolved in the spectra, UPS can reveal additional structural information for both ground and excited positive ion states of the molecule. Therefore, not only the inclusion of vibrational effects is mandatory to correctly reproduce the asymmetric bandshapes of the experimental spectra^7−9,11−14^ but also it can provide valuable information on the structural rearrangements occurring during the process and on the role of vibronic coupling.^15,16^

**Figure 1:**
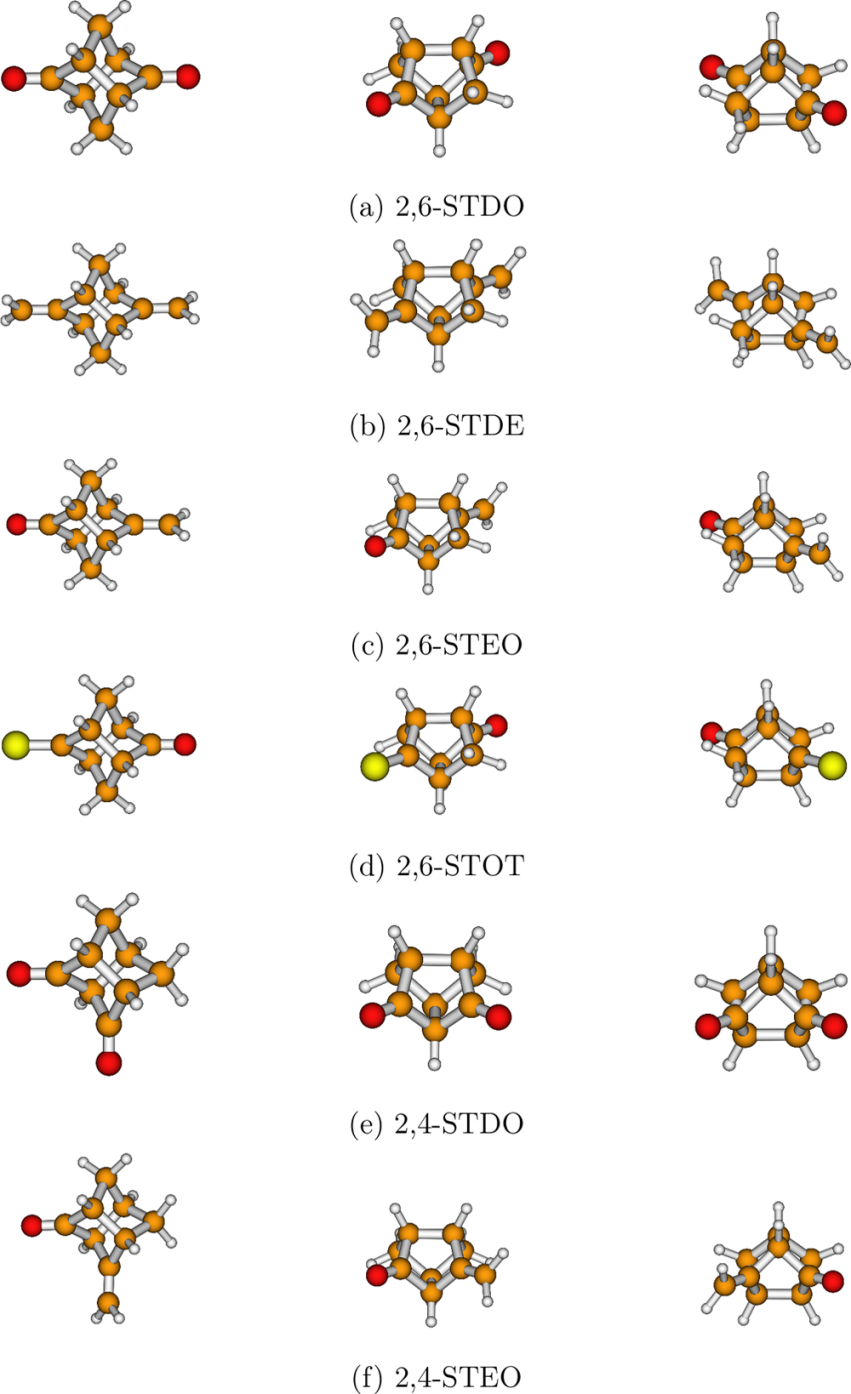
Structures of stellane molecules from three different perspectives.

In this work, assignment and computational reproduction of the UPS spectra of the six molecular systems shown in Figure 1a–f are discussed. Names, abbreviations, chirality, and symmetry point groups of these molecular systems are given in Table 1. A feature shared by various members of the stellane family, which includes the six investigated molecule, is the presence of two π-bonds separated by a rigid σ-scaffold. The same σ-scaffold allows for different orientations of the π-bonds (and of the moieties linked to the σ-scaffold through the two double bonds), giving rise to different coupling mechanisms and spectral signatures. Therefore, in our opinion, a comprehensive analysis of general trends within this class of compounds is still a topic of remarkable general interest.

**Table 1:** Names, Symmetry Point Groups, and Chirality of the Six Molecular Systems Discussed in This Work

molecule	symmetry point group	chiral?	structure
tricyclo[3.3.0.0^3,7^]octane-2,6-dione (2,6-STDO)	*D*_2_	yes	Figure 1a
2,6-dimethylenetricyclo[3.3.0^1,5^.0^3,7^]octane (2,6-STDE)	*D*_2_	yes	Figure 1b
6-methylenetricyclo[3.3.0.0^3,7^]octan-2-one (2,6-STEO)	*C*_2_	yes	Figure 1c
2-oxotricyclo[3.3.0.0^3,7^]octane-6-thione (2,6-STOT)	*C*_2_	yes	Figure 1d
tricyclo[3.3.0.0^3,7^]octane-2,4-dione (2,4-STDO)	*C*_*s*_	no	Figure 1e
4-methylenetricyclo[3.3.0.0^3,7^]octan-2-one (2,4-STEO)	*C*_1_	yes	Figure 1f

While the synthesis^17−20^ and experimental UPS spectra^20,21^ of the six compounds mentioned above are well-known, the available computational results^22^ do not take into account the vibrational signatures of the electronic transitions associated with low-energy ionizations, thus preventing a comprehensive analysis of band shapes.

Therefore, the main purposes of the present study are (i) the validation of a computational protocol combining methods based on one-electron Green’s functions for the calculation of ionization potentials (IPs) with a characterization of ground electronic states obtained with DFT-based methods and (ii) the integration of the computational results already available in the literature, especially concerning the characterization of the vibrational progressions observed in the experimental spectra.^23^

## 2 Vibrational Signatures in UPS Spectra: A Brief Overview

In the framework of the Born–Oppenheimer (BO) approximation, reliable approximations of the PESs of the electronic states involved in the electronic transition are needed to reproduce its vibrational signatures. The usefulness of methods based on one-electron Green’s functions for the calculation of vertical IPs is well recognized. However, the vertical IP corresponds to the energy difference between two PESs at a specific nuclear configuration (the equilibrium geometry of the neutral molecular system): therefore, additional information on the PESs of neutral and ionized states is needed.

In principle, methods based on one-electron Green’s functions can provide reliable approximations of PESs, and indeed, the connection between the one-electron Green’s function and the ground state energy is well-known. The first calculations of the vibrational signatures associated with electronic transitions computed with electron propagator theory-based methods can be traced back to the works of Cederbaum et al.,^24−26^ and since then many other contributions have appeared.^12,27,28^ Other approaches (not employing the electron propagator theory for the calculation of electronic transitions) have been proposed and successfully applied to the calculation of vibrationally resolved UPS spectra.^29^

Second-order many body perturbation theory (MBPT2) can be recovered from the expression of the ground state energy derived from a second-order approximation to the one-electron Green’s function, and hence the analytic gradients of the ground state energy can be obtained from the MBPT2 treatment. For what concerns the ionized states, the gradients of electron propagator poles are needed. Although the corresponding analytical expressions have been derived for the second-order approximation to the self-energy matrix^30,31^ and for some higher-order extensions,^31^ the corresponding implementations in general electronic structure codes are still lacking. Therefore, in order to reach a good compromise between computational cost and accuracy, a pragmatic approach has been employed: the PES of the neutral form of a molecular system is approximated with DFT-based methods, while the energy difference between a ionized form and the neutral form of the same molecular system is computed with methods based on the electron propagator theory.

## 3 Computational Details

All the calculations have been performed with a development version of the Gaussian suite of programs.^32^ Geometry optimization and harmonic force field evaluations of the ground (neutral) electronic state have been carried out with DFT, employing the B3LYP^33−35^ hybrid exchange-correlation functional in conjunction with the maug-cc-pVTZ basis set.^36,37^ The calculation of vertical IPs has been performed with two different approximations of the electron propagator matrix, namely the OVGF method^38−40^ (which is computationally cheap and retains a quasi-particle picture) and the NR2 method^41^ (a nondiagonal approximation, which is computationally more demanding than the OVGF method yet cheaper than other nondiagonal approaches) in conjunction with the maug-cc-pVTZ basis set.

For what concerns the calculation of the vibronic bandshapes of the first two (or three, in the case of the 2,6-STOT molecule) ionized electronic states, the time independent (TI) approach has been employed.^42^ Vibronic transitions have been computed with the Vertical Gradient (VG) model^43,44^ in conjunction with the Franck–Condon (FC) approximation.^45^ In the VG model, the derivatives of the differences of the final (ionized) and the initial (neutral) state PESs with respect to the normal coordinates of the initial state (evaluated at the equilibrium geometry of the initial state) are needed. In the vibronic calculations, the band positions (which are given by the vertical IPs) have been calculated with the NR2 approximation, and the calculation of the gradients has been performed numerically with the (computationally less demanding) OVGF method by means of an external python script, employing the following expression:
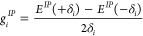
1

In eq 1, *g*_*i*_^*IP*^ is the *i*th Cartesian component of the gradient **g**^*IP*^ expressed in Cartesian coordinates, *E*^*IP*^(+δ_*i*_) and *E*^*IP*^(−δ_*i*_) are the vertical IPs calculated with displacements from the equilibrium geometry (of the initial, neutral electronic state) of, respectively, +δ and −δ along the *i*th Cartesian coordinate. In this study, the value of δ has been set equal to 0.001 Å. It must be noticed that  (and not **g**^*IP*^) is needed for the calculation of the shift vector **K** in the VG model. However, the following relationship holds
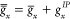
2

In eq 2, the subscript *x* indicates a generic coordinate system. The numerical differentiation is performed at the equilibrium geometry of the initial (neutral) electronic state, and therefore, **g̅** = 0: the direct consequence is that , and the calculation of the components of  can be carried out with eq 1. The coordinate system adopted for the nuclear coordinates is important: in practice, in this study, the gradient is calculated in Cartesian coordinates with eq 1 and is provided to the Gaussian software for the calculation of the shift vector **K**. In the TI approach to the calculation of vibronic spectra, three user-defined prescreening factors are employed (in order to select the most relevant FC overlap integrals):^46,47^ in this study, the values *C*_1_^*max*^ = 20, *C*_2_^*max*^ = 13, and *N*_*i*_^*max*^ = 10^8^ have been adopted (if not otherwise specified).

## 4 Results

**Figure 2:**
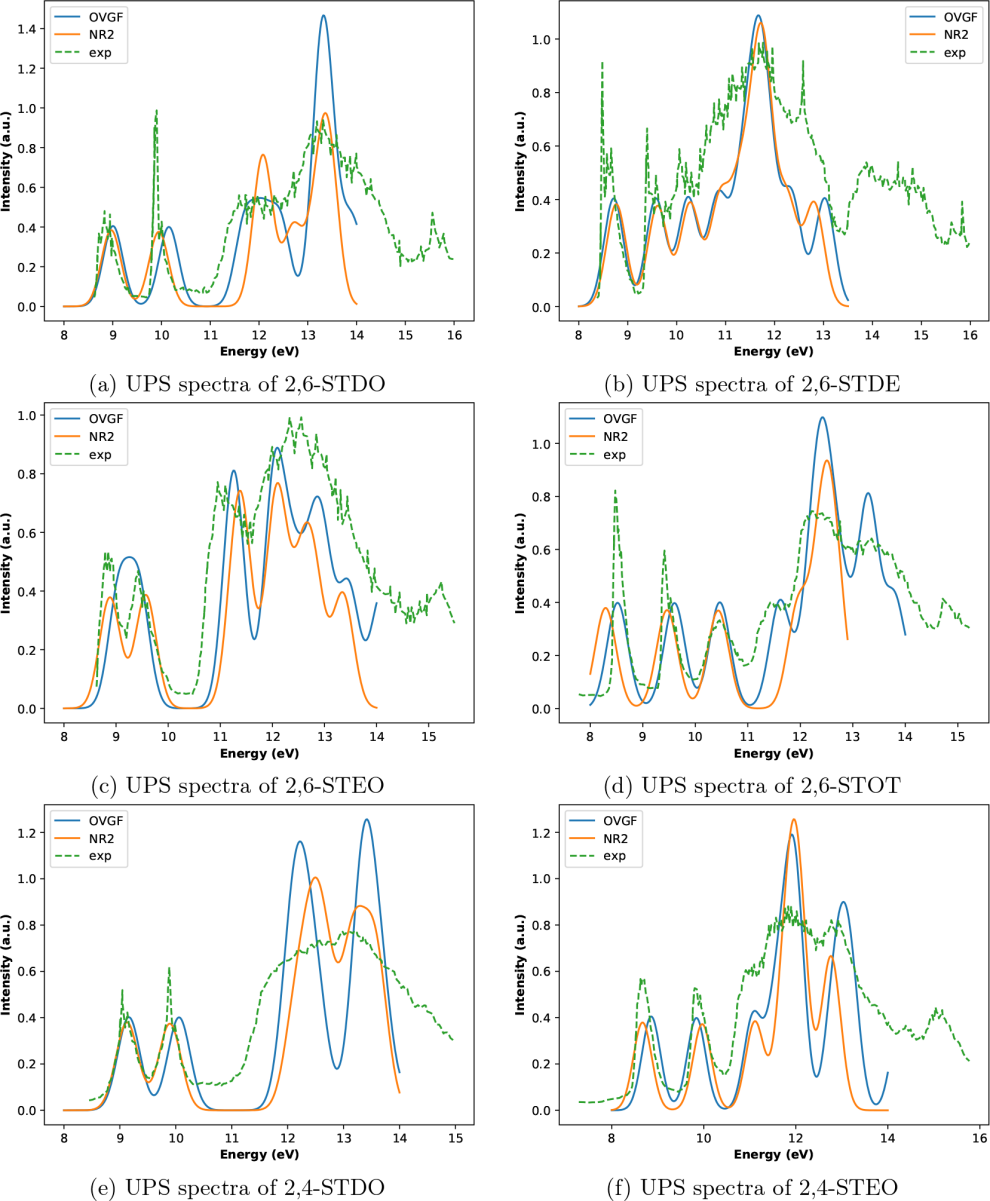
UPS spectra of the six molecular systems listed in Table 1. Intensities are given in arbitrary units, and transition energies are provided in electronvolt (eV); the experimental spectra (dashed green lines) are taken from the literature (see the text). As reported in ref (21), the sharp peak at ∼12.58 eV in panel (b) is due to the presence of water in the sample.

In what follows, the main results obtained for the six molecular systems listed in Table 1 are presented and discussed. In Figure 2, experimental UPS spectra (taken from refs (20) and (21)) are compared with simulated ones (calculated in this study, at NR2/maug-cc-pVTZ and OVGF/maug-cc-pVTZ level of theory). Intensities are given in arbitrary units, and therefore, the absolute intensities are adjusted to match their experimental counterparts. However, a comparison of the relative intensities is still possible and meaningful. Each pole of the electron propagator matrix corresponds to a transition energy, and its pole strength corresponds to the intensity of the same transition. Gaussian functions are employed to reproduce broadening effects in the computational results.

Detailed assignments of the transition energies are provided in Tables S1, S3, S5, S6, S8, and S10 (reported in the SI). The assignments proposed in this study can be compared with the assignments reported in Table 1 of ref (20) and Table 1 of ref (21). For what concerns 2,6-STDO and 2,6-STDE, the results of a previous quantum chemical study (see ref (22)) are reported in Tables S1 and S3 together with the results of our calculations.

**Figure 3:**
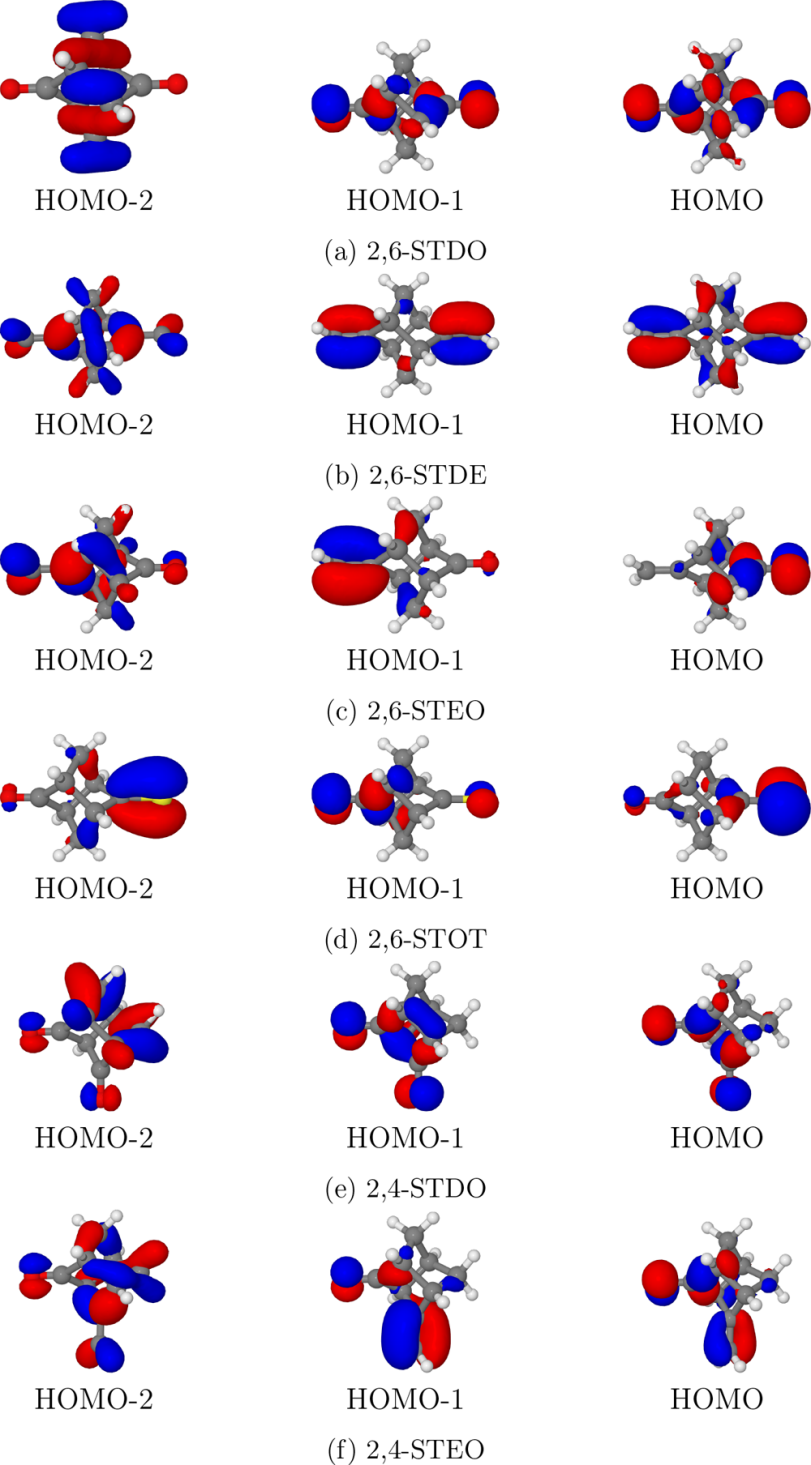
Molecular orbitals of the investigated molecules (isodensity surfaces at ±0.05 (e/bohr^3^)^1/2^).

The transition energies of UPS spectra are usually assigned to the electron binding energies of specific Molecular Orbitals (MO), i.e., the validity of the quasi-particle picture is assumed: when the diagonal approximation is adopted (as is the case of the OVGF method and of the values obtained by means of the Koopmans Theorem, KT), this assumption is valid, because Dyson orbitals (DOs) are proportional to MOs. However, when a nondiagonal approximation is employed, this assumption must be verified. In the case of a nondiagonal approximation, DOs are obtained (in general) as linear combinations of several MOs: in practice, in most cases (at least for closed-shell molecules), the linear combination is dominated by a single MO, and therefore, the transition energies can be still assigned to a specific MO. For the molecular systems investigated in this study, the transition energies obtained with the OVGF and the NR2 approximations are comparable (see Figure 2), and in the case of the (nondiagonal) NR2 approximation, each DO is dominated by a single MO (in Figure 3 outer valence MOs of the six molecule are reported). Therefore, in the following, as well as in the SI, each electronic transition energy is associated with a specific molecular orbital also for the NR2 results. However, in the tables collected in the SI, it has been reported whether other contributions (besides the contribution of the dominant MO) to a specific DO are relevant for a nondiagonal approximation.

Calculated transition energies assigned to outer valence MOs are in good agreement with the experimental values for both electron propagator methods employed in this study (see Figure 2 and the tables reported in the SI), with the exception of the 2,6-STEO molecule: in this case, the agreement of the NR2 results with the experimental values is more satisfactory than the results obtained when the OVGF approximation is employed (see Figure 2c). All the outer valence MOs (and therefore all the transition energies) are mainly (but not exclusively) related to the lone pairs of the chalcogens (oxygen and sulfur atoms) or to the π-bonds of the six compounds investigated (see Figure 3). Nevertheless, a partial delocalization of the outer valence MOs on the central σ-scaffold (which is the central molecular unit common to all the molecular systems considered in this work) is observed for MOs related to the oxygen lone pairs (for 2,4-STDO and 2,4-STEO, this effect was already recognized in ref (20)): this is the case for 7*b*_2_ and 6*b*_3_ MOs of 2,6-STDO, 12*b* MO of 2,6-STEO, 11*b* MO of 2,6-STOT, and 11*a*″ and 15*a*′ MOs of 2,4-STDO. The phenomenon can be observed also in the case of 2,4-STEO (see Table S10 in the SI), but in this specific case, the (approximate) identification of the DOs with the MOs seems particularly problematic for what concerns the outer valence MOs.

The experimental trend^20^ which suggests a coupling between the lone pairs of the oxygen atoms in 2,6-STDO stronger than the coupling observed in the case of 2,4-STDO is confirmed by both OVGF and NR2 results (see Tables S1 and S8). A good agreement between theory and experiment is found also for the transition energies associated with the outer valence MOs of 2,6-STEO and 2,4-STEO (in this case, the coupling is stronger in the case of 2,4-STEO molecule). All the assignments proposed in refs (20) and (21) are confirmed: this is not surprising, because only the first, well-separated experimental bands were assigned in refs (20) and (21); moreover, a diagonal approximation to the electron propagator matrix provides results which are even quantitatively in agreement with the experimental values in almost all the cases considered in this study. However, it must be noticed that corrections to the KT results are needed in order to correctly reproduce (even qualitatively) the experimental results. For example, KT does not provide reliable results for the first transitions of 2,6-STEO and 2,6-STOT (see Tables S5 and S6), as was already recognized in refs (20) and (21).

As mentioned above, the matching between the experimental values and the OVGF results for the transition energies of the outer valence MOs (13*b* and 12*b*) of the 2,6-STEO molecular system improves when the NR2 approximation is adopted (see Figure 2c). Since the corresponding DOs are dominated by contributions of the 13*b* and 12*b* MOs (see Table S5) when the NR2 approximation is employed, the importance of the nondiagonal contributions has been verified as follows: single point calculations (at the equilibrium geometry) with the diagonal counterpart of the NR2 approximation (the so-called P3 method)^48^ and its renormalized extension (P3+, renormalized partial third-order method)^49^ have been carried out (employing the maug-cc-pVTZ basis set), and the difference between the transition energies associated with the 13*b* and 12*b* MOs obtained at P3/maug-cc-pVTZ and P3+/maug-cc-pVTZ levels of theory is compared with the same difference obtained at the OVGF/maug-cc-pVTZ and NR2/maug-cc-pVTZ levels. For the P3 method, the difference is 0.51 eV, while the OVGF and NR2 results are 0.38 and 0.69 eV, respectively: these values suggest that the discrepancy between experimental and calculated transition energies observed for the 2,6-STEO molecule can be removed even within the diagonal approximation. This interpretation is further confirmed by the P3+ result, which is expected to have the best combination of accuracy and efficiency within the diagonal self-energy approximation.^50^ Indeed, the P3+ energy difference is 0.63 eV which is lower than the NR2 result by only 0.06 eV.

The results sketched in Figure 2 and reported in Tables S1, S3, S5, S6, S8, and S10 suggest a good agreement between experimental and calculated results for electron binding energies lower than about 14 eV. For what concerns the six compounds studied, in most cases, the NR2 and OVGF approximations provide very similar results (in the case of the 2,6-STOT molecule, the OVGF results are even closer to the experimental results than the NR2 ones).

**Figure 4:**
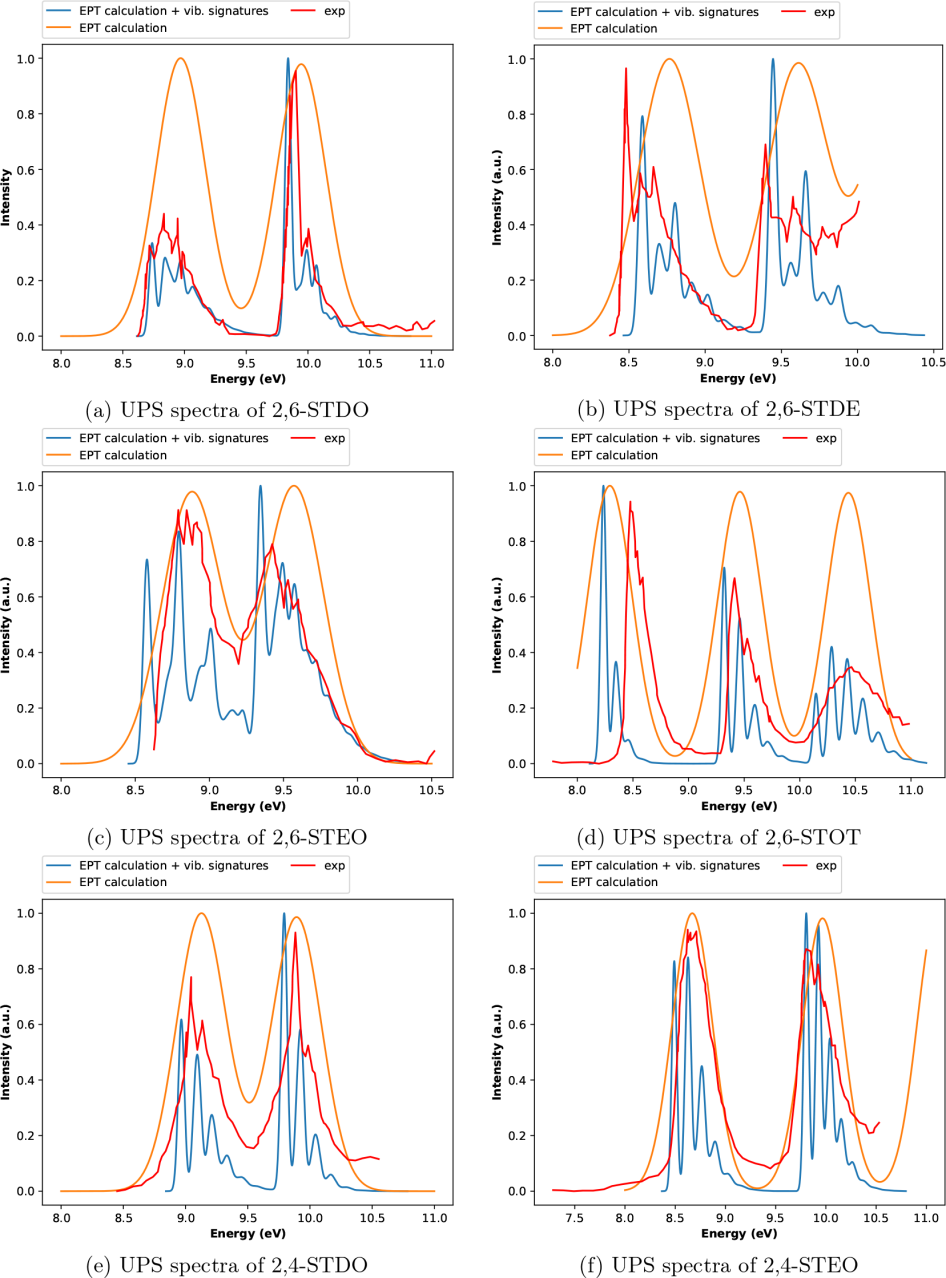
Vibronic UPS spectra of the six compounds listed in Table 1. The experimental spectra (continuous red lines) are taken from the literature (see the text).

Next, the vibronic structure has been computed for the lower electron binding energies, for which well-separated vibronic structures are available from refs (20) and (21). The results reported in Figure 4 show that, in spite of the limited resolution of the experimental spectra provided in refs (20) and (21), a fairly good agreement is obtained between experimental and computational results with the exception of the vibronic structures of the 2,6-STEO molecule.

The assignment of the most intense vibronic transitions can be found in the SI. For what concerns the five compounds whose experimental spectra are in good agreement with our calculations, the most intense vibronic transition is always the |0⟩ → |0⟩ one. This is particularly evident for the second electronic transition of the 2,6-STDO molecule and was already recognized in ref (21).

For what concerns 2,6-STDO and 2,6-STDE, vibronic signatures characterized by intense |0⟩ → |0⟩ vibronic transitions (see Tables S2 and S4) and a computational extrapolation of the final state geometry based on the VG model suggest that the equilibrium geometries of the excited cationic states are very similar to those of the ground electronic states of the neutral molecules. Moreover, the good agreement between experimental and calculated results (particularly evident in the case of 2,6-STDO) gives further support to the reliability of our computational approach.

Other intense vibronic features for the electronic transitions of interest of the molecules 2,6-STDO and 2,6-STDE are associated with the normal modes depicted in Figures S1 and S2. The vibronic transitions associated with the symmetric stretching of the two double bonds are of particular interest: despite the limited resolution of the available experimental spectra, the calculated spectra of the 2,6-STDE molecule support the assignment of the vibronic features at 8.7 eV and at 9.6 eV to the vibronic transition |0⟩ → |47(1)⟩ (see Figure 4b, Table S4, and Figure S2e); moreover, the results listed in Table S4 (and plotted in Figure 4b) suggest the possibility of a direct observation of the vibronic transitions associated with the first overtone (|0⟩ → |47(2)⟩) of the symmetric stretching of the two double bonds in the 2,6-STDE molecule. To verify this hypothesis, new high-resolution UPS spectra would be especially welcome. Both double bonds involve a carbon and an oxygen atom in the case of 2,6-STDO and two carbon atoms in the case of 2,6-STDE; the corresponding normal modes are the 40th normal mode in the case of 2,6-STDO (see Figure S1e) and the 47th normal mode in the case of 2,6-STDE (see Figure S2e). The other intense vibronic transitions listed in Tables S2 and S4 involve fundamental transitions of CH bendings (see Figures S1b–d and S2b–d) of the central σ-scaffold with two exceptions, namely the |0⟩ → |8(1)⟩ vibronic transition for the 2,6-STDO molecule (because a skeletal deformation is involved in the 8th normal mode, see Figure S1a) and the |0⟩ → |7(1)⟩ transition for the 2,6-STDE molecule. In this latter case, the CH bendings of the peripheral CH_2_ units are involved in the 7th normal mode, see Figure S2a).

In the cases of 2,6-STOT, 2,4-STDO, and 2,4-STEO, the computational protocol successfully employed for 2,6-STDO and 2,6-STDE leads to extrapolated equilibrium geometries (obtained in the framework of the VG model) for the ionized states which differ substantially from the equilibrium geometry of the neutral ground state. The relevant changes in the equilibrium geometries are accompanied (not surprisingly) by small FC overlap integrals for the |0⟩ → |0⟩ vibronic transitions: in the presence of such large-amplitude displacements, a better approximation of the final PES is needed to obtain a reliable simualtion of vibronic effects. This is achieved, for instance, by adopting other harmonic models for the description of the final state PES^51,52^ or with a computational approach suitable for the description of vibronic transitions in flexible molecular systems.^53^ In practice, another route (computationally less demanding) can be employed, which is based on the combination of the computational protocol described in section 3 with a reduced-dimensionality scheme: in this case, the vibronic calculation is carried out on a subset of the normal modes of the molecular system of interest, while the contributions of the other normal modes are neglected. In this study, the selection of the normal modes included in the vibronic calculation is based on the inspection of the components of the shift vector **K**: too high values of the low-frequency components of **K** are avoided by neglecting the contributions of the corresponding normal modes in the vibronic calculation. For what concerns the computational protocol, all the normal modes with a fundamental frequency below a user-defined value are neglected in the vibronic calculation. To ensure that the full spectrum is described at the same level of approximation, the same subset of normal modes is used for all the electronic transitions. Despite its usefulness, this protocol can lead to the exclusion of some normal modes, which could be safely included in the vibronic calculation referred to as a specific electronic transition: this can be due to a normal mode with a higher fundamental frequency which does not allow a proper description (in the framework of the VG model) of the vibrational signature of another electronic transition of the same molecular system. In order to ensure the reproducibility of the computational results, the user-defined values employed for the calculations presented in this study are provided in the SI.

The reduced-dimensionality model leads to results for 2,6-STOT, 2,4-STDO, and 2,4-STEO molecules in good agreement with their experimental UPS spectra (see Figure 4d–f), and the extrapolated equilibrium geometries for the ionized states are very similar to the equilibrium geometries of the corresponding neutral ground states. In the case of the 2,4-STEO molecule, the extrapolated equilibrium structures (for the two ionized states of interest) for the fragment C(*sp*^2^)=C(*sp*^2^)H_2_ (which is planar in the neutral ground state) are slightly bent: this is consistent with the assignment of a partial π_*CC*_ character for the outer valence MOs 26*a* and 25*a* (proposed in Table S10).

Although (as mentioned above) the |0⟩ → |0⟩ vibronic transition is the most intense one for the three vibronic structures of 2,6-STOT investigated in this study, the vibronic band associated with the |0⟩ → |0⟩ vibronic transition is not necessarily the most intense of the vibronic structures calculated (and observed) for a certain electronic transition. More specifically, in Figure 4d, the vibronic band which corresponds to |0⟩ → |0⟩ transition is the most intense one for the vibronic structures of the first (between 8 and 9 eV) and the second (between 9 and 10 eV) electronic transitions, but the same is not true for the vibronic structure of the third electronic transition (between 10 and 11 eV): indeed, a single vibronic band can result from the contributions of several vibronic transitions, as a consequence of the finite bandwidth of each vibronic transition. Besides the |0⟩ → |0⟩ transition, the other most relevant contributions (identified in Table S7) involve the bending of CH bonds of the central σ-scaffold (see Figure S3a–i) together with the CO (Figure S3k) and CS (Figure S3j) stretchings. The relative intensity of the |0⟩ → |0⟩ transitions and the vibrational progressions for the three vibronic structures reported in Figure 4d and assigned in Table S7 are consistent with the assigments proposed in Table S6 for the three electronic transitions: the less intense |0⟩ → |0⟩ transition pertains to the electronic transition assigned to the removal of one electron from the π_*CS*_ MO, while the other two vibronic transitions of interest are assigned to *n*_*O*_ and *n*_*S*_ MOs. Moreover, the 11*b* MO is partially delocalized on the σ-scaffold: this is consistent with a vibronic progression which is not dominated by (and almost reduced to) the |0⟩ → |0⟩ transition.

The vibronic structures computed for both the outer valence ionization transitions of 2,4-STDO are similar: the first (and most intense) vibronic band (assigned to the |0⟩ → |0⟩ transition) is followed by vibronic bands of decreasing intensity (the assignment is provided in Table S9). These vibronic structures are similar to those calculated for the ionization of the 11*b* MO of 2,6-STOT. The contribution to the first vibronic structure of the vibronic transitions associated with the symmetric (see Figure S4j) and the antisymmetric (see Figure S4i) CO stretchings is relevant as well (see Table S9). The other intense vibronic transitions (besides the |0⟩ → |0⟩) are assigned to the CH bendings of the central σ-scaffold, see Figure S4a–h.

For what concerns the 2,4-STEO molecule, the assignments of vibronic structures are provided in Table S11. Besides the |0⟩ → |0⟩ transition and the transitions involving the CH bendings of the central σ-scaffold, an intense band associated with the CC stretching (Figure S5n) is observed as well.

A tentative explanation of the disagreement between experimental and computational results in the case of 2,6-STEO (see Figure 4c) is the following one. At first glance, the VG model seems to work well (the extrapolated equilibrium geometries for the ionized states are very similar to the equilibrium geometry of the ground state of the neutral molecular system), while the absolute intensities of the vibronic bands are significantly lower than their counterparts in the other computed spectra discussed in this work; this is not clear from the spectra displayed in Figure 4, because the intensities are reported in arbitrary units (i.e., only the relative intensities in the same spectrum can be compared). Therefore, a first attempt to go beyond the FC approximation retaining the first-order contributions to the transition properties (the so-called HT term)^54^ was done, without significant improvements in the computed results. Probably the agreement between computational and experimental results can be improved employing other harmonic models (for example, the adiabatic Hessian, AH, model) and avoiding the approximation **J** = **I**: an attempt in this direction would benefit from the implementation of analytic gradients for the cationic states’ PES. We expect that anharmonic effects for the ionized states do not play a major role for semirigid molecules like 2,6-STEO. The unsatisfactory agreement between experiment and purely electronic calculations at the OVGF/maug-cc-pVTZ level (see Figure 2c and Table S5) casts severe doubts about the reliability of the OVGF method for the calculation of gradients. As mentioned above, the discrepancy between experimental and computational OVGF/maug-cc-pVTZ results does not imply (at least in principle) the failure of the diagonal approximation to the electron propagator matrix. In order to verify whether the partial inclusion of third-order corrections may be responsible for the observed discrepancies, we simulated the vibronic spectrum with gradients calculated at the D2/maug-cc-pVTZ level. The D2 method includes only second-order corrections to the diagonal elements of the electron propagator matrix. Nevertheless, the agreement between computational and experimental results remains poor.

## 5 Conclusions

In this work, the UPS spectra of six semirigid molecules were simulated with the inclusion of vibrational modulation effects by means of a computational strategy recently proposed by some of the present authors.^23^ Concerning the electronic computations, good agreement between computational and experimental ionization energies is obtained by both diagonal and nondiagonal approaches. Only in one case (2,6-STEO moelcule), the nondiagonal renormalized second-order (NR2) results are significantly more accurate than their OVGF counterparts. Within the diagonal approximation, this discrepancy can be reduced if more accurate methods as the renormalized partial third-order approximation (P3+) are employed. Vibrational modulation effects were then accounted for by a composite scheme, using the NR2 model for vertical ionization energies, the OVGF method for the nuclear energy gradients of the cationic states’ PES, and hybrid density functionals for harmonic frequencies and normal modes of the initial (neutral) state. These data were then used to compute vibronic band shapes by the vertical gradient model within a time-independent framework. A general reduced dimensionality scheme allowed for extending the simulations from semirigid to flexible molecules involving a reduced number of weakly coupled large amplitude motions. For all but one molecule, the protocol reproduced the experimental spectra with remarkable accuracy, and in particular, the different band-shapes of the low binding transitions are well reproduced. In this connection, further analysis of the remaining problematic case is surely needed by both computational (e.g., implementation of NR2 analytic gradients^31^ for the ionized states) and experimental (recording of a more resolved UPS spectrum) points of view. However, we think that the results of the present study provide a general and reliable picture of the interplay of different stereoelectronic factors in determining the physical-chemical properties of an interesting class of cage compounds and point out the non-negligible role of vibrational modulation effects in tuning the overall spectroscopic outcome.

## Supporting Information Available

For each molecule: energies and assignment of electronic transitions; energies, intensities, and assignment of main vibronic transitions; and graphical representation of normal modes (PDF)
