# Supplementary material for: Interplay of Stereoelectronic and Vibrational Modulation Effects in Tuning the UPS Spectra of Unsaturated Hydrocarbon Cage Compounds
Source: J Chem Theory Comput. 2020 Jul 15;16(8):5218–26. doi: 10.1021/acs.jctc.0c00645 (PMC8009518; doi:10.1021/acs.jctc.0c00645)
Supplement: Supplementary file 1 — ct0c00645_si_001.pdf [file ct0c00645_si_001.pdf]

Supporting Information: Interplay of stereo-electronic and vibrational modulation effects in tuning the UPS spectra of unsaturated hydrocarbon cage compounds

Lorenzo Paoloni,† Marco Fusè,† Alberto Baiardi,‡ and Vincenzo Barone*,†

†*Scuola Normale Superiore, Piazza dei Cavalieri 7, 56125, Pisa, Italy*

‡*Lab. für Physikalische Chemie, Vladimir-Prelog -Weg 1-5/10, 8093 Zürich, Switzerland*

E-mail: vincenzo.barone@sns.it

1 Assignment of UPS spectra

In what follows, computational and experimental values of transition energies for the six UPS spectra showed and discussed in the main article are provided. For what concerns the assignment of the purely electronic transition energies, the quasiparticle picture is retained. The numeration of MOs do not take into account the core levels. The pole strength are provided in parenthesis. Values of the transition energies calculated by means of the OVGF method are provided for transition energies below 20 eV, while values calculated with the NR2 approximation are given only for transitions assigned to outer valence MOs. In the cases of 2,6-STDO (table S1) and 2,6-STDE (table S3), the computational results provided in ref. S1 are also reported.

The assignment of the vibronic transitions is carried out for five of the six compounds studied in this work. The analysis is not carried out for the 2,6-STEO molecule due to the

distance between experimental and calculated values; in other words, in this case the analysis of the computed values do not have a counterpart in the real world and therefore is left out. Normal modes associated with the most intense vibronic transitions are depicted.

Only the most intense vibronic transitions are listed, although in most of the cases many other vibronic transitions should be taken into account in order to completely reproduce the vibronic signature of a specific electronic transition.

A reduced-dimensinality scheme has been employed for the calculation of vibronic transitions of 2,6-STOT, 2,4-STDO and 2,4-STEO. The protocol is based on the exclusion (from the vibronic calculation) of all the normal modes with a fundamental frequency below a user-defined threshold. These thresholds are 800 cm^{-1} in the case of 2,4-STDO and 850 cm^{-1} in the cases of 2,6-STOT and 2,4-STEO.

Table S1: Values and assignment of each electronic transition for 2,6-STDO molecule.

transition	method					experimental (UPS)
	KT (HF/maug-cc-pVTZ)	OVGF/maug-cc-pVTZ	OVGF/cc-pVTZ[1]	NR2/maug-cc-pVTZ	ADC(3)/cc-pVDZ[1]	
$7b_2$ [n (+σ)]	10.31	9.01 (0.899)	8.96 (0.901)	8.97 (0.876)	8.94 (0.897)	8.84[3]
$6b_3$ [n (+σ)]	11.61	10.15 (0.894)	10.09 (0.896)	9.95 (0.866)[2]	10.02 (0.889)	9.90[3]
7a [σ]	13.05	11.70 (0.905)	11.64 (0.907)	12.06 (0.886)	11.92 (0.906)	
$6b_2$ [σ(+n)]	13.13	12.07 (0.893)	11.99 (0.894)	12.11 (0.864)	11.94 (0.883)	} 11.5-12.5[4]
$6b_1$ [σ]	13.80	12.45 (0.904)	12.40 (0.906)	12.70 (0.885)	12.62 (0.904)	
$5b_3$ [π]	14.39	13.49 (0.883)	13.36 (0.882)	13.21 (0.840)	13.11 (0.854)	
$5b_2$ [σ(+n)]	14.47	13.29 (0.897)	13.20 (0.899)	13.18 (0.874)[2]	13.16 (0.891)	
$4b_2$ [σ]	14.80	13.27 (0.902)	13.21 (0.904)	13.44 (0.873)[2]	13.40 (0.893)	} 13-14[4]
6a [σ]	15.03	13.29 (0.895)	13.20 (0.898)	13.41 (0.871)	13.41 (0.894)	
$5b_1$ [σ]	15.29	13.91 (0.906)	13.85 (0.907)	13.92 (0.883)	13.89 (0.901)	
$4b_3$ [σ(+n)]	16.17	14.48 (0.885)	14.37 (0.888)	-	14.27 (0.850)	} 14-14.5[4]
$3b_2$ [π]	16.42	14.71 (0.878)	14.58 (0.882)	-	14.48 (0.669)	
$3b_3$ [σ]	17.27	15.65 (0.893)	15.57 (0.893)	-	15.75 (0.811)	} 15.5[5]
$4b_1$ [σ]	18.11	16.09 (0.884)	15.99 (0.883)	-	15.96 (0.867)	
$2b_2$ [σ(+n)]	18.91	17.07 (0.883)	16.97 (0.883)	-	17.16 (0.761)	
5a [σ]	19.42	17.32 (0.881)	17.24 (0.880)	-	17.24 (0.828)	
$3b_1$ [σ]	20.13	18.02 (0.883)	17.96 (0.882)	-	18.02 (0.836)	
4a [σ]	20.58	18.43 (0.882)	18.36 (0.881)	-	18.41 (0.852)	

[1] taken from ref. S1

[2] slight contributions from other MOs

[3] taken from ref. S2

[4] our assignment

[5] taken from ref. S2; assignment proposed in ref. S1

Table S2: Energies, intensities and assignment of the main vibronic transitions for the first and the second bands of the spectrum of 2,6-STDO molecule.

transition	main contributions	energy (eV)	intensity (a. u.)
$7b_2$ [n $(+\sigma)$]	$\|0\rangle \rightarrow \|0\rangle$	8.737	$0.769 \cdot 10^{-3}$
	$\|0\rangle \rightarrow \|12(1)\rangle$	8.834	$0.518 \cdot 10^{-3}$
	$\|0\rangle \rightarrow \|24(1)\rangle$	8.874	$0.181 \cdot 10^{-3}$
	$\|0\rangle \rightarrow \|33(1)\rangle$	8.893	$0.188 \cdot 10^{-3}$
	$\|0\rangle \rightarrow \|40(1)\rangle$	8.967	$0.262 \cdot 10^{-3}$
	$\|0\rangle \rightarrow \|12(1), 40(1)\rangle$	9.064	$0.177 \cdot 10^{-3}$
$6b_3$ [n $(+\sigma)$]	$\|0\rangle \rightarrow \|0\rangle$	9.839	$0.205 \cdot 10^{-2}$
	$\|0\rangle \rightarrow \|8(1)\rangle$	9.915	$0.250 \cdot 10^{-3}$
	$\|0\rangle \rightarrow \|33(1)\rangle$	9.995	$0.425 \cdot 10^{-3}$
	$\|0\rangle \rightarrow \|40(1)\rangle$	10.069	$0.402 \cdot 10^{-3}$

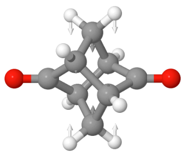

(a) 8-th normal mode

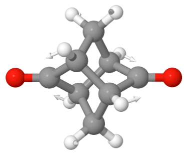

(b) 12-th normal mode

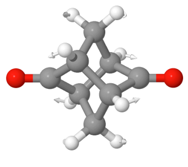

(c) 24-th normal mode

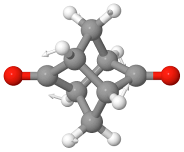

(d) 33-th normal mode

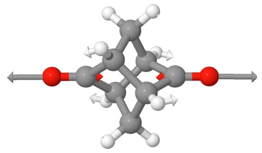

(e) 40-th normal mode

Figure S1: Graphical representation of the normal modes of 2,6-STDO, numbered with respect to the associated fundamental frequency in ascending order; only the normal modes involved in the most intense vibronic transitions are reported (see table S2).

Table S3: Values and assignment of each electronic transition for 2,6-STDE molecule.

transition	method					experimental (UPS)
	KT (HF/maug-cc-pVTZ)	OVGF/maug-cc-pVTZ	OVGF/cc-pVTZ[1]	NR2/maug-cc-pVTZ	ADC(3)/cc-pVDZ[1]	
$7b_2$ [π]	9.06	8.71 (0.898)	8.69 (0.899)	8.77 (0.874)	8.68 (0.885)	8.49[2]
$6b_3$ [π]	9.98	9.58 (0.896)	9.54 (0.897)	9.61 (0.866)	9.55 (0.879)	9.40[2]
$6b_2$ [σ]	11.35	10.23 (0.898)	10.20 (0.902)	10.27 (0.876)	10.32 (0.897)	10.05[2]
7a [σ]	11.96	10.84 (0.897)	10.81 (0.901)	10.87 (0.874)	10.96 (0.897)	10.9[3]
$5b_3$ [σ]	12.54	11.32 (0.897)	11.28 (0.901)	11.28 (0.875)	11.38 (0.896)	} 11.4[3]
$6b_1$ [σ]	12.81	11.63 (0.897)	11.59 (0.900)	11.59 (0.874)	11.70 (0.896)	
$5b_2$ [σ]	13.03	11.90 (0.896)	11.86 (0.899)	11.82 (0.870)	11.95 (0.890)	} 11.9[3]
6a [σ]	13.20	11.66 (0.891)	11.63 (0.895)	11.76 (0.868)	11.84 (0.893)	
$4b_2$ [σ]	13.72	12.35 (0.898)	12.32 (0.902)	12.27 (0.875)	12.41 (0.897)	} 12.5[4]
$5b_1$ [σ]	14.29	13.02 (0.898)	13.00 (0.900)	12.81 (0.873)	12.97 (0.892)	
$4b_3$ [σ]	15.57	14.29 (0.882)	14.25 (0.884)	-	14.12 (0.847)	} 14-15[4]
$3b_2$ [σ]	15.79	14.52 (0.876)	14.49 (0.878)	-	14.31 (0.805)	
$3b_3$ [σ]	16.29	14.86 (0.884)	14.81 (0.887)	-	14.79 (0.710)	
$4b_1$ [σ]	16.82	15.11 (0.887)	15.07 (0.887)	-	15.13 (0.871)	
$2b_2$ [σ]	17.65	15.91 (0.877)	15.86 (0.877)	-	16.01 (0.549)	15.9[4]
5a [σ]	18.52	16.70 (0.876)	16.66 (0.876)	-	16.60 (0.700)	
$3b_1$ [σ]	19.19	17.19 (0.875)	17.16 (0.875)	-	17.25 (0.736)	
4a [σ]	19.56	17.54 (0.874)	17.50 (0.874)	-	17.54 (0.785)	

[1] taken from ref. S1
[2] taken from ref. S2
[3] taken from ref. S2; assignment proposed in ref. S1
[4] our assignment

Table S4: Energies, intensities and assignment of the main vibronic transitions for the first and the second bands of the spectrum of 2,6-STDE molecule.

transition	main contributions	energy (eV)	intensity (a. u.)
$7b_2$ [π]	$\|0\rangle \rightarrow \|0\rangle$	8.588	$0.148 \cdot 10^{-1}$
	$\|0\rangle \rightarrow \|16(1)\rangle$	8.688	$0.367 \cdot 10^{-2}$
	$\|0\rangle \rightarrow \|31(1)\rangle$	8.728	$0.189 \cdot 10^{-2}$
	$\|0\rangle \rightarrow \|47(1)\rangle$	8.803	$0.698 \cdot 10^{-2}$
	$\|0\rangle \rightarrow \|16(1), 47(1)\rangle$	8.903	$0.173 \cdot 10^{-2}$
	$\|0\rangle \rightarrow \|47(2)\rangle$	9.019	$0.164 \cdot 10^{-2}$
$6b_3$ [π]	$\|0\rangle \rightarrow \|0\rangle$	9.445	$0.168 \cdot 10^{-1}$
	$\|0\rangle \rightarrow \|7(1)\rangle$	9.503	$0.178 \cdot 10^{-2}$
	$\|0\rangle \rightarrow \|23(1)\rangle$	9.559	$0.257 \cdot 10^{-2}$
	$\|0\rangle \rightarrow \|47(1)\rangle$	9.660	$0.879 \cdot 10^{-2}$
	$\|0\rangle \rightarrow \|23(1), 47(1)\rangle$	9.774	$0.134 \cdot 10^{-2}$
	$\|0\rangle \rightarrow \|47(2)\rangle$	9.876	$0.229 \cdot 10^{-2}$

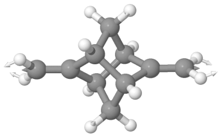

(a) 7-th normal mode

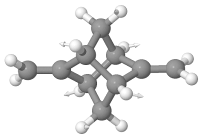

(b) 16-th normal mode

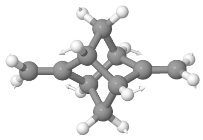

(c) 23-th normal mode

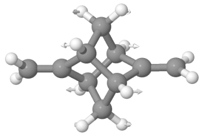

(d) 31-th normal mode

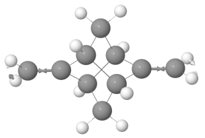

(e) 47-th normal mode

Figure S2: Graphical representation of the normal modes of 2,6-STDE, numbered with respect to the associated fundamental frequency in ascending order; only the normal modes involved in the most intense vibronic transitions are reported (see table S4).

Table S5: Values and assignment of each electronic transition for 2,6-STEO molecule.

transition	method			experimental (UPS)[1]
	KT (HF/maug-cc-pVTZ)	OVGF/maug-cc-pVTZ	NR2/maug-cc-pVTZ	
13b [π]	9.85	9.44 (0.900)	9.57 (0.878)	9.40[3]
12b [n(+σ)]	10.33	9.06 (0.895)	8.88 (0.868)	8.85[3]
13a [σ]	12.48	11.25 (0.901)	11.43 (0.880)	} 10.95[3]
11b [σ(+n)]	12.55	11.27 (0.899)	11.32 (0.876)[2]	
10b [n(+σ)]	13.14	12.07 (0.893)	12.04 (0.861)	} 12-13.5[4]
12a [σ]	13.29	12.02 (0.900)	12.13 (0.879)	
9b [π(+σ)]	14.02	13.00 (0.888)	12.72 (0.858)	
11a [σ]	14.05	12.42 (0.894)	12.56 (0.870)	
8b [σ]	14.23	12.78 (0.900)	12.81 (0.867)[2]	
10a [σ]	14.79	13.47 (0.901)	13.36 (0.878)	
7b [π(+σ)]	15.73	14.10 (0.882)	-	
6b [σ(+n)]	16.17	14.83 (0.877)	-	
5b [σ(+n)]	16.75	15.14 (0.890)	-	15.1[4]
9a [σ]	17.48	15.61 (0.885)	-	
4b [σ]	18.28	16.48 (0.880)	-	
8a [σ]	18.96	16.99 (0.878)	-	
7a [σ]	19.64	17.59 (0.879)	-	
6a [σ]	20.05	17.97 (0.878)	-	

[1] taken from ref. S2
[2] slight contributions from other MOs
[3] assignemnt proposed in ref. S2
[4] our assignment

Table S6: Values and assignment of each electronic transition for 2,6-STOT molecule.

transition	method			experimental (UPS)[1]
	KT (HF/maug-cc-pVTZ)	OVGF/maug-cc-pVTZ	NR2/maug-cc-pVTZ	
13b[n_S]	9.17	8.52 (0.892)	8.29 (0.87)	8.49[3]
12b[π_{CS}]	10.72	10.47 (0.894)	10.44 (0.859)	10.50[3]
11b[$n_O(+\sigma)$]	11.00	9.60 (0.891)	9.46 (0.861)[2]	9.45[3]
12a[σ]	13.04	11.62 (0.899)	11.96 (0.875)	11.40[3]
11a[σ]	13.58	12.21 (0.895)	12.40 (0.868)[2]	12-13[4]
10b[$\pi_{CO}(+\sigma)$]	13.61	12.44 (0.889)	12.49 (0.818)[2]	
10a[σ]	13.93	12.45 (0.896)	12.68 (0.872)[2]	
9b[$\sigma(+n_O)$]	14.22	12.78 (0.89)	-	
8b[$\pi_{CO}(+\sigma)$]	14.67	13.32 (0.887)	-	13-14.5[4]
7b[$\sigma(+n_O)$]	14.96	13.26 (0.888)	-	
9a[σ]	15.29	13.80 (0.900)	-	
6b[$\pi_{CO}(+\sigma)$]	16.22	14.45 (0.88)	-	
8a[σ]	16.82	15.05 (0.874)	-	15[4]
5b[$\sigma(+n_O)$]	17.23	15.52 (0.889)	-	
4b[σ]	18.75	16.85 (0.874)	-	
7a[σ]	19.01	16.87 (0.868)	-	
6a[σ]	20.08	17.91 (0.876)	-	
5a[σ]	20.51	18.33 (0.875)	-	

[1] taken from ref. S3
[2] slight contributions from other MOs
[3] assignment proposed in ref. S3
[4] our assignment

Table S7: Energies, intensities and assignment of the main vibronic transitions for the first, the second and the third bands of the spectrum of 2,6-STOT molecule.

transition	main contributions	energy (eV)	intensity (a. u.)
$13b[n_S]$	$\|0\rangle \rightarrow \|0\rangle$	8.253	$0.269 \cdot 10^{-3}$
	$\|0\rangle \rightarrow \|16(1)\rangle$	8.362	$0.133 \cdot 10^{-4}$
	$\|0\rangle \rightarrow \|21(1)\rangle$	8.374	$0.116 \cdot 10^{-4}$
	$\|0\rangle \rightarrow \|23(1)\rangle$	8.387	$0.138 \cdot 10^{-4}$
	$\|0\rangle \rightarrow \|27(1)\rangle$	8.395	$0.107 \cdot 10^{-4}$
$12b[\pi_{CS}]$	$\|0\rangle \rightarrow \|0\rangle$	10.146	$0.502 \cdot 10^{-4}$
	$\|0\rangle \rightarrow \|19(1)\rangle$	10.259	$0.125 \cdot 10^{-4}$
	$\|0\rangle \rightarrow \|27(1)\rangle$	10.288	$0.182 \cdot 10^{-4}$
	$\|0\rangle \rightarrow \|28(1)\rangle$	10.289	$0.111 \cdot 10^{-4}$
	$\|0\rangle \rightarrow \|37(1)\rangle$	10.316	$0.115 \cdot 10^{-4}$
$11b[n_O(+\sigma)]$	$\|0\rangle \rightarrow \|0\rangle$	9.321	$0.154 \cdot 10^{-3}$
	$\|0\rangle \rightarrow \|21(1)\rangle$	9.441	$0.232 \cdot 10^{-4}$
	$\|0\rangle \rightarrow \|24(1)\rangle$	9.458	$0.351 \cdot 10^{-4}$
	$\|0\rangle \rightarrow \|29(1)\rangle$	9.470	$0.142 \cdot 10^{-4}$
	$\|0\rangle \rightarrow \|35(1)\rangle$	9.481	$0.136 \cdot 10^{-4}$
	$\|0\rangle \rightarrow \|40(1)\rangle$	9.550	$0.127 \cdot 10^{-4}$

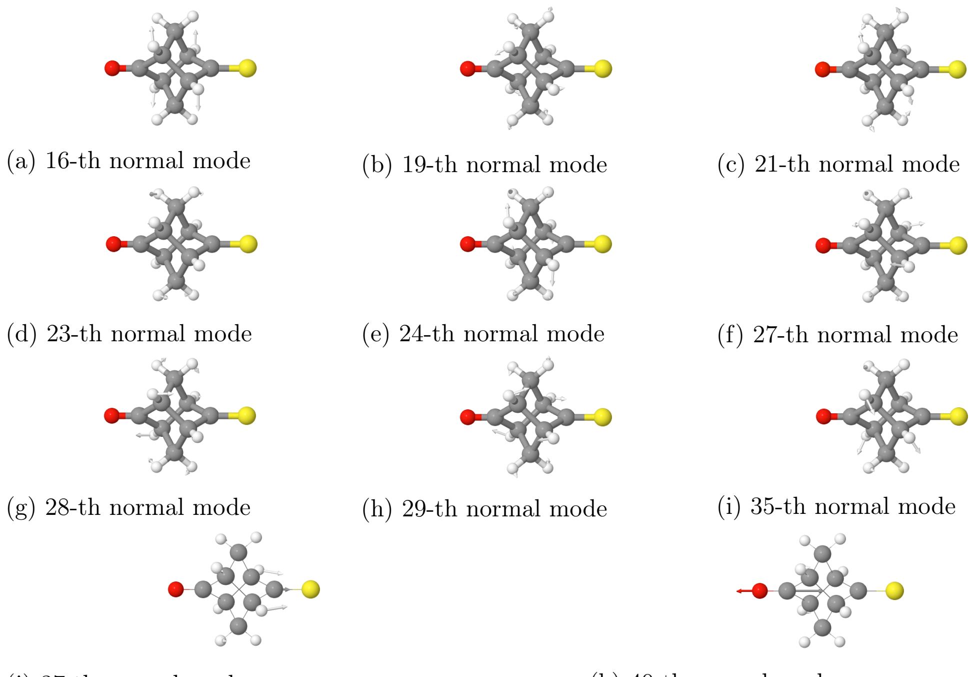

(a) 16-th normal mode (b) 19-th normal mode (c) 21-th normal mode

(d) 23-th normal mode (e) 24-th normal mode (f) 27-th normal mode

(g) 28-th normal mode (h) 29-th normal mode (i) 35-th normal mode

(j) 37-th normal mode (k) 40-th normal mode

Figure S3: Graphical representation of the normal modes of 2,6-STOT, numbered with respect to the associated fundamental frequency in ascending order; only the normal modes involved in the most intense vibronic transitions are reported (see table S7).

Table S8: Values and assignment of each electronic transition for 2,4-STDO molecule.

transition	method			experimental (UPS)[2]
	KT (HF/maug-cc-pVTZ)	OVGF/maug-cc-pVTZ	NR2/maug-cc-pVTZ	
11a″[$n(+\sigma)$]	10.51	9.16 (0.896)	9.13 (0.870)	9.04[3]
15a′[$n(+\sigma)$]	11.47	10.06 (0.895)	9.89 (0.864)	9.88[3]
10a″[$\sigma(+n)$]	13.28	12.03 (0.900)	12.14 (0.874)	12-14[4]
14a′[$\sigma(+n)$]	13.44	12.19 (0.901)	12.41 (0.872)[1]	
13a′[σ]	13.64	12.28 (0.902)	12.52 (0.879)[1]	
9a″[$\sigma(+n)$]	14.01	12.54 (0.900)	12.73 (0.877)	
8a″[π]	14.43	13.38 (0.885)	13.17 (0.836)	
12a′[$n(+\sigma)$]	14.55	13.28 (0.893)	13.16 (0.820)[1]	
11a′[$\sigma(+n)$]	14.80	13.45 (0.899)	13.49 (0.850)[1]	
7a″[σ]	15.00	13.69 (0.902)	13.62 (0.863)[1]	
10a′[$\pi(+\sigma)$]	16.18	14.47 (0.884)	-	
6a″[$\pi(+\sigma)$]	16.83	14.95 (0.876)	-	
9a′[σ]	17.76	15.91 (0.887)	-	
5a″[σ]	17.96	15.98 (0.884)	-	
8a′[σ]	18.76	16.94 (0.885)	-	
7a′[σ]	19.62	17.61 (0.883)	-	
4a″[σ]	20.48	18.18 (0.874)	-	
6a′[σ]	20.51	18.35 (0.880)	-	

[1] slight contributions from other MOs
[2] taken from ref. S3
[3] assignment proposed in ref. S3
[4] our assignment

Table S9: Energies, intensities and assignment of the main vibronic transitions for the first and the second bands of the spectrum of 2,4-STDO molecule.

transition	main contributions	energy (eV)	intensity (a. u.)
$11a''[n(+\sigma)]$	$\|0\rangle \rightarrow \|0\rangle$	8.966	$0.201 \cdot 10^{-1}$
	$\|0\rangle \rightarrow \|16(1)\rangle$	9.076	$0.597 \cdot 10^{-2}$
	$\|0\rangle \rightarrow \|20(1)\rangle$	9.084	$0.129 \cdot 10^{-2}$
	$\|0\rangle \rightarrow \|22(1)\rangle$	9.095	$0.160 \cdot 10^{-2}$
	$\|0\rangle \rightarrow \|25(1)\rangle$	9.105	$0.411 \cdot 10^{-2}$
	$\|0\rangle \rightarrow \|29(1)\rangle$	9.113	$0.133 \cdot 10^{-2}$
	$\|0\rangle \rightarrow \|33(1)\rangle$	9.124	$0.113 \cdot 10^{-2}$
	$\|0\rangle \rightarrow \|39(1)\rangle$	9.192	$0.105 \cdot 10^{-2}$
	$\|0\rangle \rightarrow \|40(1)\rangle$	9.197	$0.175 \cdot 10^{-2}$
	$\|0\rangle \rightarrow \|16(1); 25(1)\rangle$	9.215	$0.122 \cdot 10^{-2}$
$15a'[n(+\sigma)]$	$\|0\rangle \rightarrow \|0\rangle$	9.793	$0.297 \cdot 10^{-1}$
	$\|0\rangle \rightarrow \|15(1)\rangle$	9.902	$0.711 \cdot 10^{-2}$
	$\|0\rangle \rightarrow \|22(1)\rangle$	9.922	$0.109 \cdot 10^{-2}$
	$\|0\rangle \rightarrow \|25(1)\rangle$	9.932	$0.631 \cdot 10^{-2}$
	$\|0\rangle \rightarrow \|27(1)\rangle$	9.934	$0.359 \cdot 10^{-2}$
	$\|0\rangle \rightarrow \|15(1); 25(1)\rangle$	10.041	$0.151 \cdot 10^{-2}$

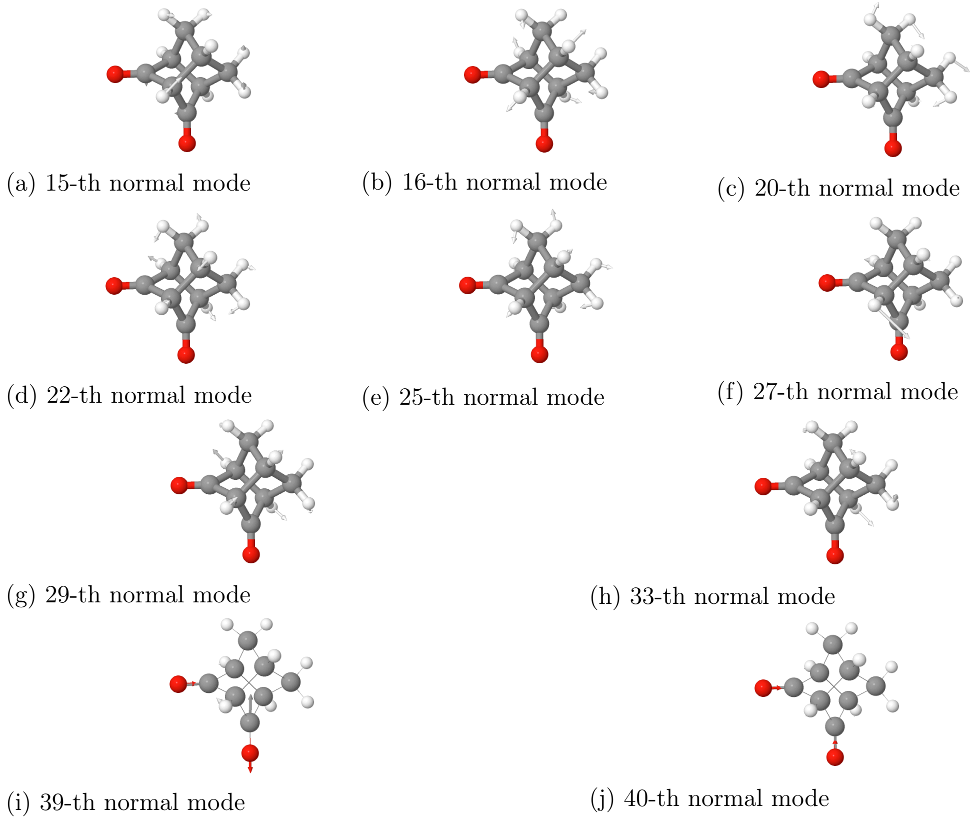

(a) 15-th normal mode
(b) 16-th normal mode
(c) 20-th normal mode
(d) 22-th normal mode
(e) 25-th normal mode
(f) 27-th normal mode
(g) 29-th normal mode
(h) 33-th normal mode
(i) 39-th normal mode
(j) 40-th normal mode

Figure S4: Graphical representation of the normal modes of 2,4-STDO, numbered with respect to the associated fundamental frequency in ascending order; only the normal modes involved in the most intense vibronic transitions are reported (see table S9).

Table S10: Values and assignment of each electronic transition for 2,4-STEO molecule.

transition	method			experimental (UPS)[4]
	KT (HF/maug-cc-pVTZ)	OVGF/maug-cc-pVTZ	NR2/maug-cc-pVTZ	
26a[$\pi_{CC}(+n+\sigma)$]	9.52	8.86 (0.899)	8.67 (0.870)[1]	8.65[5]
25a[$n(+\pi_{CC}+\sigma)$]	10.94	9.84 (0.892)	9.97 (0.862)[2]	9.77[5]
24a[$\sigma(+n)$]	12.22	11.07 (0.897)	11.11 (0.873)[3]	10.90[5]
23a[σ]	12.85	11.56 (0.899)	11.75 (0.878)	
22a[σ]	13.08	11.83 (0.900)	11.91 (0.878)[3]	} 11.5-12.5[6]
21a[σ]	13.20	12.01 (0.898)	12.03 (0.872)[3]	
20a[σ]	13.41	11.98 (0.897)	12.09 (0.874)	
19a[$\pi_{CO}(+\sigma)$]	14.09	13.04 (0.885)	12.70 (0.829)[3]	
18a[σ]	14.20	12.82 (0.900)	12.82 (0.838)[3]	} 12.5-13.5[6]
17a[σ]	14.47	13.21 (0.902)	-	
16a[$\pi_{CO}(+\sigma)$]	15.84	14.26 (0.880)	-	
15a[$\sigma(+n)$]	16.42	14.91 (0.873)	-	
14a[σ]	16.95	15.28 (0.889)	-	
13a[σ]	17.33	15.44 (0.884)	-	
12a[σ]	18.18	16.41 (0.883)	-	
11a[σ]	19.10	17.18 (0.880)	-	
10a[σ]	19.86	17.67 (0.869)	-	
9a[σ]	19.98	17.90 (0.877)	-	

[1] relevant contribution from MO 25a
[2] relevant contribution from MO 26a
[3] slight contributions from other MOs
[4] taken from ref. S3
[5] assignment proposed in ref. S3
[6] our assignment

Table S11: Energies, intensities and assignments of the main vibronic transitions for the first and the second bands of the spectrum of 2,4-STEO molecule.

transition	main contributions	energy (eV)	intensity (a. u.)
$26a[\pi_{CC}(+n+\sigma)]$	$\|0\rangle \to \|0\rangle$	8.491	$0.372 \cdot 10^{-1}$
	$\|0\rangle \to \|18(1)\rangle$	8.602	$0.193 \cdot 10^{-2}$
	$\|0\rangle \to \|21(1)\rangle$	8.605	$0.426 \cdot 10^{-2}$
	$\|0\rangle \to \|24(1)\rangle$	8.612	$0.244 \cdot 10^{-2}$
	$\|0\rangle \to \|25(1)\rangle$	8.620	$0.570 \cdot 10^{-2}$
	$\|0\rangle \to \|26(1)\rangle$	8.626	$0.394 \cdot 10^{-2}$
	$\|0\rangle \to \|27(1)\rangle$	8.628	$0.410 \cdot 10^{-2}$
	$\|0\rangle \to \|28(1)\rangle$	8.631	$0.710 \cdot 10^{-2}$
	$\|0\rangle \to \|29(1)\rangle$	8.634	$0.362 \cdot 10^{-2}$
	$\|0\rangle \to \|34(1)\rangle$	8.643	$0.309 \cdot 10^{-2}$
	$\|0\rangle \to \|37(1)\rangle$	8.651	$0.154 \cdot 10^{-2}$
	$\|0\rangle \to \|39(1)\rangle$	8.654	$0.247 \cdot 10^{-2}$
$25a[n(+\pi_{CC}+\sigma)]$	$\|0\rangle \to \|0\rangle$	9.805	$0.390 \cdot 10^{-1}$
	$\|0\rangle \to \|17(1)\rangle$	9.914	$0.153 \cdot 10^{-2}$
	$\|0\rangle \to \|21(1)\rangle$	9.919	$0.142 \cdot 10^{-1}$
	$\|0\rangle \to \|23(1)\rangle$	9.924	$0.449 \cdot 10^{-2}$
	$\|0\rangle \to \|24(1)\rangle$	9.926	$0.106 \cdot 10^{-1}$
	$\|0\rangle \to \|27(1)\rangle$	9.942	$0.287 \cdot 10^{-2}$
	$\|0\rangle \to \|34(1)\rangle$	9.957	$0.227 \cdot 10^{-2}$
	$\|0\rangle \to \|43(1)\rangle$	10.019	$0.526 \cdot 10^{-2}$
	$\|0\rangle \to \|21(2)\rangle$	10.033	$0.260 \cdot 10^{-2}$
	$\|0\rangle \to \|21(1);23(1)\rangle$	10.038	$0.164 \cdot 10^{-2}$
	$\|0\rangle \to \|21(1);24(1)\rangle$	10.040	$0.389 \cdot 10^{-2}$
	$\|0\rangle \to \|24(2)\rangle$	10.047	$0.145 \cdot 10^{-2}$
	$\|0\rangle \to \|21(1);43(1)\rangle$	10.133	$0.192 \cdot 10^{-2}$
	$\|0\rangle \to \|24(1);43(1)\rangle$	10.140	$0.144 \cdot 10^{-2}$

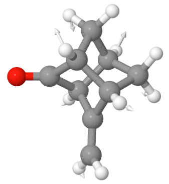
(a) 17-th normal mode

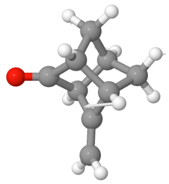
(b) 18-th normal mode

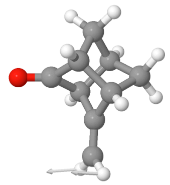
(c) 21-th normal mode

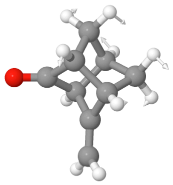
(d) 23-th normal mode

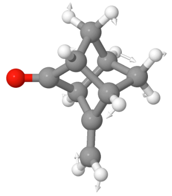
(e) 24-th normal mode

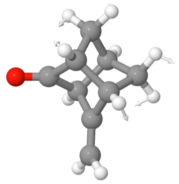
(f) 25-th normal mode

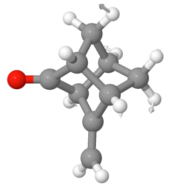
(g) 26-th normal mode

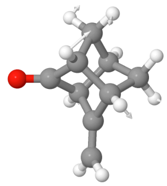
(h) 27-th normal mode

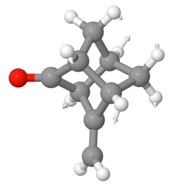
(i) 28-th normal mode

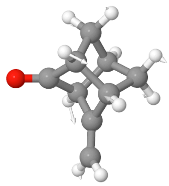
(j) 29-th normal mode

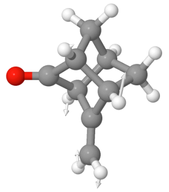
(k) 34-th normal mode

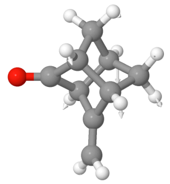
(l) 37-th normal mode

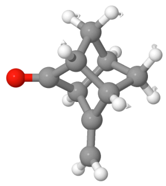
(m) 39-th normal mode

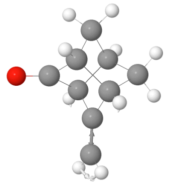
(n) 43-th normal mode

Figure S5: Graphical representation of the normal modes of 2,4-STEO, numbered with respect to the associated fundamental frequency in ascending order; only the normal modes involved in the most intense vibronic transitions are reported (see table S11).

References

(S1) Knippenberg, S.; François, J.-P.; Deleuze, M. S. Green's function study of the one-electron and shake-up ionization spectra of unsaturated hydrocarbon cage compounds. *J. Comput. Chem.* **2006**, *27*, 1703–1722.

(S2) Gleiter, R.; Lange, H.; Borzyk, O. Photoelectron Spectra, Ab Initio SCF MO, and Natural Bond Orbital Studies on Stellenes. Long-Range π/σ Interactions. *J. Am. Chem. Soc.* **1996**, *118*, 4889–4895.

(S3) Gleiter, R.; Gaa, B.; Sigwart, C.; Lange, H.; Borzyk, O.; Rominger, F.; Irngartinger, H.; Oeser, T. Preparation and Properties of Stelladiones. *Eur. J. Org. Chem.* **1998**, *1998*, 171–176.
